# Phenology and phylogeny of *Hyalomma* spp. ticks infesting one-humped camels (*Camelus dromedarius*) in the Tunisian Saharan bioclimatic zone

**DOI:** 10.1051/parasite/2021038

**Published:** 2021-05-18

**Authors:** Khawla Elati, Faten Bouaicha, Mokhtar Dhibi, Boubaker Ben Smida, Moez Mhadhbi, Isaiah Obara, Safa Amairia, Mohsen Bouajila, Barbara Rischkowsky, Mourad Rekik, Mohamed Gharbi

**Affiliations:** 1 Laboratoire de Parasitologie, Univ. Manouba, Institution de la Recherche et de l’Enseignement Supérieur Agricoles, École Nationale de Médecine Vétérinaire de Sidi Thabet 2020 Sidi Thabet Tunisia; 2 Commissariat Régional de Développement Agricole (CRDA) 3200 Tataouine Tunisia; 3 Institute for Parasitology and Tropical Veterinary Medicine, Freie Universität Berlin Robert-von-Ostertag-Str. 7–13 14163 Berlin Germany; 4 International Centre for Agricultural Research in the Dry Areas (ICARDA) P.O. Box 5689 Addis Ababa Ethiopia; 5 International Center for Agricultural Research in the Dry Areas (ICARDA) P.O. Box 950764 Amman 11195 Jordan

**Keywords:** Ticks, Camels, *Hyalomma*, Phenology, Phylogeny, Tunisia

## Abstract

In this study, we report the results of a survey of *Hyalomma* ticks infesting one-humped camels in southern Tunisia. Examinations were conducted every second or third month on 406 camels in Tataouine district from April 2018 to October 2019. A total of 1902 ticks belonging to the genus *Hyalomma* were collected. The ticks were identified as adult *H. impeltatum* (41.1%; *n* = 782), *H. dromedarii* (32.9%; *n* = 626), *H. excavatum* (25.9%; *n* = 493), and *H. marginatum* for a single specimen. Although the camels were infested by ticks throughout the year, the highest overall infestation prevalence was observed in April 2018 (*p* < 0.01). The overall infestation intensity varied between 2.7 and 7.4 ticks/animal. There were no statistically significant differences in tick infestation prevalence based on age categories of the camels, and the overall infestation prevalence was between 82.7% and 97.4%. Female camels were significantly more infested with ticks (88.3%) than males (65.5%) (*p* < 0.01). The infestation prevalence of camels varied significantly according to the region where sampling took place (*p* < 0.01), but no correlations were found with abiotic factors. The preferred attachment sites for adult *Hyalomma* ticks were the sternum (38.3%; *n* = 729/1902), around the anus (36.2%; *n* = 689/1902), udder (18.4%; *n* = 350/1902), and inner thigh (6.9%; *n* = 132/1902). Morphological classification of ticks was corroborated by sequencing the cytochrome c oxidase I (*Cox1*) and 16S rDNA genes, and these sequences were also used to infer phylogenetic relationships. A single *H. dromedarii* seemed to be a natural hybrid with *H. rufipes*. More attention should be devoted by the veterinary services to the infestation of camels by ticks.

## Abstract (fr)

Dans cette étude, les résultats d’une enquête concernant les tiques *Hyalomma* infestant les dromadaires dans le sud de la Tunisie sont présentés. Des examens ont été menés tous les deux ou trois mois sur 406 dromadaires dans le district de Tataouine entre avril 2018 et octobre 2019. Au total, 1902 tiques appartenant au genre *Hyalomma* ont été collectées. Les tiques adultes ont été identifiées comme *H. impeltatum* (41,1 % ; *n* = 782), *H. dromedarii* (32,9 % ; *n* = 626), *H. excavatum* (25,9 % ; *n* = 493) et un seul spécimen de *H. marginatum*. Bien que les dromadaires aient été infestés par les tiques tout au long de l’année, la prévalence globale d’infestation la plus élevée a été observée en avril 2018 (*p* < 0,01). L’intensité globale d’infestation variait entre 2,7 et 7,4 tiques/animal. Il n’y avait pas de différence statistiquement significative de l’infestation par les tiques en fonction des catégories d’âge des dromadaires, et la prévalence globale d’infestation se situait entre 82,7 et 97,4 %. Les dromadaires femelles étaient significativement plus infestés par les tiques (88,3 %) que les mâles (65,5 %) (*p* < 0,01). La prévalence d’infestation variait significativement en fonction des régions (*p* < 0.01) mais il n’y avait pas de corrélation avec les facteurs abiotiques. Les sites de fixation préférés des tiques *Hyalomma* adultes étaient le sternum (38,3 % ; *n* = 729/1902), autour de l’anus (36,2 % ; *n* = 689/1902), la mamelle (18,4 % ; *n* = 350/1902) et la face interne de la cuisse (6,9 % ; *n* = 132/1902). La classification morphologique a été corroborée par le séquençage des gènes de l’ADNr du cytochrome c oxydase I (*Cox1*) et du 16S, et ces séquences ont également été utilisées pour déduire les relations phylogénétiques. Un seul spécimen de *H. dromedarii* semblait être un hybride naturel avec *H. rufipes*. Une plus grande attention doit être accordée par les services vétérinaires quant à l’infestation des dromadaires par les tiques.

## Introduction

Production of one-humped camels, *Camelus dromedarius* (Mammalia: Camelidae), is the principal economic activity in the far south of Tunisia, dominating all other agricultural activities outside the oasis system. In addition to arthropod infestations, several diseases are known to affect one-humped camels’ health. These include parasitic infestations (trypanosomoses [33], toxoplasmosis [39], coccidioses [16], helminthiases [44]), bacterial infections (brucellosis [23, 53] and Q-fever [7]), and viral infections (Middle East Respiratory Syndrome [56]). However, most production losses are known to result from tick infestation [46]. Ticks (Acari: Ixodidae) belonging to the genus *Hyalomma,* such as *Hyalomma dromedarii* Koch, 1844, *Hyalomma impeltatum* Schulze & Schlottke, 1930 and *Hyalomma excavatum* Koch, 1844 are the main species infesting one-humped camels in the extensive production systems of south Tunisia [47]. Where there is cohabitation with other domestic animal species, camels are also known to be infested with *Rhipicephalus* ticks [21, 24, 31, 47, 52].

*Hyalomma dromedarii* is common in northern, eastern and western Africa, in the Middle East, and in central and southern Asia [6, 52]. This species can behave as a one-, two- or three-host tick depending on the environmental conditions and host availability [3, 21, 52] and seems to be present all year round in some areas [17, 21, 25]. Camels represent the main host for adult *H. dromedarii*, but this tick species has also been collected from cattle cohabitating with one-humped camels in central and southern Tunisia [9, 31]. Although *H. dromedarii* is predominantly found in desert areas, it has also been collected from camels in the semi-arid areas of northern Tunisia (Mohamed Aziz Darghouth, personal communication). Besides camels, *H. dromedarii* juveniles also feed on rodents [52]. It is a vector of *Coxiella burnetii*, *Theileria annulata*, *Rickettsia aeschlimannii*, *R. africae, R. helvetica*, and *Babesia* spp. [4, 14, 26, 48]. Like other *Hyalomma* ticks, *H. dromedarii* is a vector of Crimean-Congo haemorrhagic fever virus (CCHFv) [12, 27].

Adult *Hyalomma excavatum* infests a variety of herbivores including small ruminants, cattle, equines and camels. This species can behave as a two- and three-host tick and its juvenile stages feed on small mammals. It is a vector of *Babesia occultans* [41], *Theileria annulata* [43], CCHFv, *Rickettsia aeschlimannii* [1] and *Coxiella burnetii* [1]. It is widely distributed in North Africa [52], particularly in Tunisia where it occurs in different bioclimatic zones [10, 40] from humid to Saharan climate (BWh climate according to the Köppen-Geiger climate classification [35]) and is collected throughout the year with a peak in spring [10].

Adult *H. impeltatum* infest mainly camels but also cattle, and the immature stages infest rodents [10]. Seasonal activity and abundance are similar to what has been reported for *H. dromedarii* [10, 52]. Its vector capacity has not been investigated in Tunisia, but it was reported to be an experimental vector of *T. annulata* [34, 52]. Other tick species such as *Rhipicephalus sanguineus* s.l., which are not specific to camels, have also been collected from camels in Tunisia [21, 24].

From previous studies on one-humped camel ticks in Tunisia, the following gaps are apparent: (i) The tick population dynamics are not fully understood as most of the studies were not carried out for extended periods of time, (ii) there is insufficient information on the behaviour of some life stages and oviposition sites, and (iii) morphology-based species identification is often not validated and/or complemented by sequence-based genotyping.

The present study aimed at addressing some of these gaps in relation to tick infestation of camels in south Tunisia. In particular, we studied the phenology of tick species infesting one-humped camels within their natural habitat in the Sahara, and possible links between tick density and environmental parameters. We also used mitochondrial cytochrome c oxidase (Cox1) and 16S rDNA sequence data to validate the morphology-based species identification and to infer phylogenetic relationships.

## Materials and methods

### Ethics statement

Ethical concerns were taken into account by adhering to local animal welfare regulations and practices and study conduct conformed to the ethical guidelines for animal usage in research of the National School of Veterinary Medicine of Sidi Thabet (Tunisia) and the *Association Tunisienne des Sciences des Animaux de Laboratoire* (ATSAL, Tunisia). Animals were handled with the permission of the regional public veterinarians at the *Commissariat Régional au Développement Agricole* (CRDA) in Tataouine and in the presence of herd owners. Interventions were restricted to manual tick removal and blood was collected by a licensed veterinarian.

### Study area

**Figure 1:**
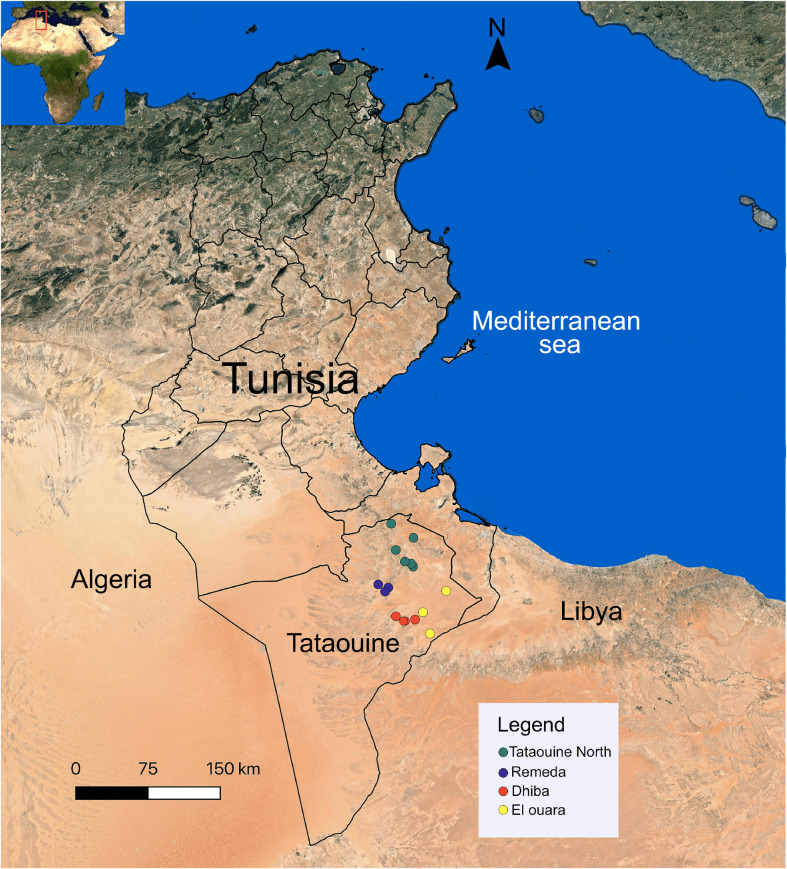
Geographic location of the tick collection sites in southern Tunisia.

The study was conducted in the Tataouine district of southern Tunisia covering a total area of 38,889 km^2^ with a Saharan climate (BWh type according to the Köppen-Geiger climate classification) (Fig. 1). Mean annual temperature is 19 °C and mean annual rainfall is 144 mm with very large interannual variations (Climate-data.org). The vegetation is characterized by the presence of xerophyte plants such as *Retama raetam, Anthyllis henoniana, Haloxylon schmittianum, Stipa tenacissima* and *Stipagrostis pungens* [54]. Livestock in Tataouine consists of one-humped camels (10,000 heads of the Maghrebi breed), small ruminants (546,870 heads of goats and 248,360 heads of sheep) and 480 heads of cattle [51]. These animals are mostly kept in extensive production systems characterized by rotational use of natural rangeland mainly between El Ouara (South-East) and Dhaher (South–West). The camels are transhumant: they move to Dhaher from March to November and to El Ouara from December to late February for watering, mating and calving. These dates may not be exactly the same for all camel owners. All samples were collected in Tataouine district, namely in Dhiba, Tataouine North, Remeda and El Ouara. It was not possible to collect samples from the Dhaher region due to difficulty of access and because we had to rely on the localization of herds by the regional veterinary services. Also, it was not possible to determine where the animals were infested by ticks because the herds were continuously on the move.

**Figure 2:**
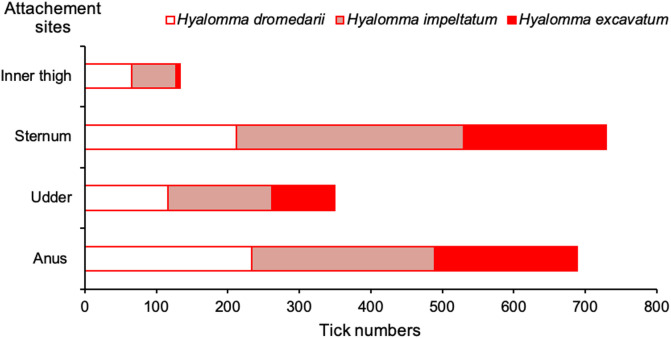
Numbers of adult *Hyalomma* ticks collected from different body parts of one-humped camels in southern Tunisia from April 2018 to October 2019.

**Figure 3:**
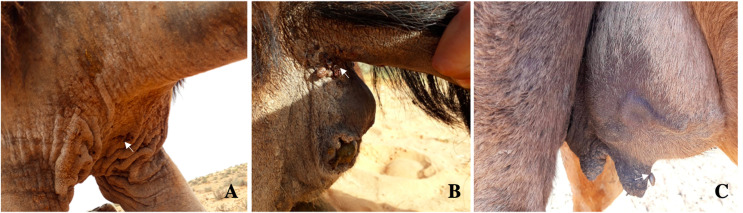
*Hyalomma* adult ticks (white rows) fixed to the sternum (A), the anus (B), and the udder (C) of camels.

### Animals, tick sampling criteria and meteorological data collection

The investigated one-humped camels belonged to the Maghrebi breed. Every second or third month from April 2018 to October 2019, herds of camels (randomly chosen at each visit) were surveyed for tick infestation. The size of herds varied between 30 and 70 individuals. A total of 406 camels were sampled, composed mainly of females (92.8%; *n* = 377). The sampled animals were divided into 6 age groups (less than 2 years, between 2 and 4 years, between 5 and 10 years, between 11 and 15 years, between 16 and 20 years, and older than 20 years). The animals were treated once a year in summer or autumn against ectoparasites using cypermethrin (Dectrol EC50^®^, Medivet, Soliman – Tunisia).

**Figure 4:**
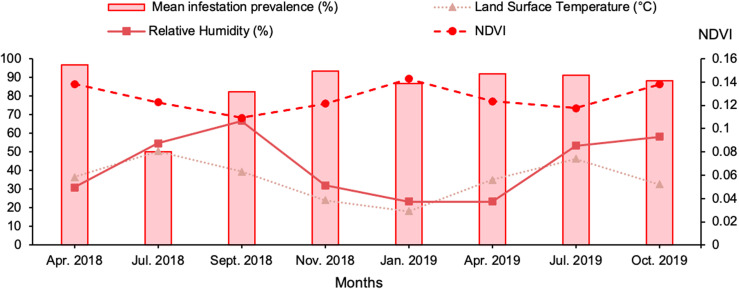
Abiotic characteristics of the studied localities. NDVI = normalized difference vegetation index.

Animals were thoroughly examined for attached ticks that were manually removed and placed in 70% ethanol. Attachment sites of the collected ticks and GPS coordinates of each sampling site were also recorded. Collected ticks were identified based on morphological criteria in the laboratory under a stereomicroscope according to Walker’s description [52]. Additionally, data collected included abiotic factors that were obtained from the ICARDA GIS unit (Fig. 4). The normalized difference vegetation index (NDVI) was extracted from the “MOD13Q1.006 Terra Vegetation Indices 16-Day Global 250 m” dataset (https://developers.google.com/earth-engine/datasets/catalog/MODIS_006_MOD13Q1#citations). The land surface temperature (LST) was obtained from the “MOD11A2.006 Terra Land Surface Temperature and Emissivity 8-Day Global 1 km” dataset (https://developers.google.com/earth-engine/datasets/catalog/MODIS_006_MOD11A2#citations).

The relative humidity data were obtained from the “GLDAS-2.1: Global Land Data Assimilation System” dataset (https://developers.google.com/earth-engine/datasets/catalog/NASA_GLDAS_V021_NOAH_G025_T3H#citations).

### DNA extraction and PCR

To study the genetic diversity of collected *Hyalomma* spp., DNA was extracted from ticks with a Wizard^®^ Genomic DNA purification kit (Promega, Madison, WI, USA), following the manufacturer’s instructions. Previously published primers were used to amplify both the 16S rDNA and Cox1 gene of *Hyalomma* spp. [2].

PCR reactions were performed in a final volume of 30 μL consisting of 1× PCR buffer, 2.5 mM MgSO_4_, 0.5 mM dNTP, 0.5 mM of each primer and 1.5 units Taq polymerase. Three μL of DNA was amplified using the following thermal profile: one denaturation cycle at 94 °C for 5 min, 35 cycles at 94 °C for 1 min each, 30 s at 45 °C and 1 min at 72 °C, and final extension at 72 °C for 10 min. Amplicons were run on a 1.5% agarose gel and visualized with ethidium bromide under ultraviolet light.

### Sequence analysis and phylogenetic inference

We amplified the Cox1 gene and 16S rDNA from 42 and 54 *Hyalomma* ticks, respectively and subjected the amplicons to bidirectional Sanger sequencing. Sequence editing, variant calling, and BLAST analysis were performed using Geneious Prime software [28]. Phylogenetic inference from the mitochondrial Cox1 sequences data and the 16S rDNA was based on maximum-likelihood analysis. Our dataset for phylogenetic inference additionally included 10 sequences representing Cox1 haplotypes distributed across the north African/Asian region. The *Rhipicephalus sanguineus* s.l. Cox1 sequence was used as the outgroup (GenBank accession number: MK820031.1). Nucleotide substitution model selection was based on the Akaike Information Criterion corrected for small sample size (AICc). Likelihood calculations for all the models were performed with PhyML_3.0_linux6 [22]. Akaike weights were used as evidence that a given model was the best for the data. All model evaluation steps described above were implemented in jModelTest 2.1.10 [37]. Maximum-likelihood tree-search algorithms were generated in PAUP 4.0 beta version using the parameter estimates for the best-fit model identified, as described above [50]. We also calculated branch support using 1000 bootstrap replicates.

### Statistical analyses

The results were expressed using two parasitological indicators [11]:

Infestationprevalence(%)=100×(numberofinfestedcamels/numbersofexaminedcamels)

Infestationintensity =numberofticks/numbersofinfestedcamels

Abundance =numberofticks/numbersofexaminedcamels

A Chi-square test was performed using SPSS software (version 21, IBM, USA) [45] to study the monthly variation in collected tick numbers. The Pearson correlation coefficient was estimated to determine the degree of correlation between tick numbers and studied environmental factors, i.e., relative humidity, land surface temperature, altitude, and NDVI values. For all these tests, a 5% threshold value was set for significance.

## Results

### Monthly variation in indicators of tick infestation

Throughout the whole sampling period, only *Hyalomma* ticks were collected (*n* = 1902); tick male-to-female sex ratio was 1.2, 0.48 and 1.5 for *H. dromedarii*, *H. impeltatum* and *H. excavatum*, respectively. The dominant tick species was *H. impeltatum* (41.1%; *n* = 782), followed by *H. dromedarii* (32.9%; *n* = 626) and *H. excavatum* (25.9%; *n* = 493). Only one specimen of *H. marginatum* (*p* < 0.01) was found in January.

The preferred attachment sites of *Hyalomma* ticks were the sternum (38.3%; *n* = 729/1902) and anus (36.2%; *n* = 689/1,902) (Figs. 3A and 3B), followed by the udder (adult female camels) (Fig. 3C) (18.4%; *n* = 350/1902) and inner thigh of the hind legs (6.9%; *n* = 132/1902) (*p* < 0.01). As shown in Figure 2, the sternum and anus were the most preferred attachment body parts for the three tick species. *Hyalomma excavatum* ticks were distributed equally between the sternum (40.3%) and the anus (40.5%); 40.6% of *H. impeltatum* were collected from the sternum and 32% from the anus; 37.2% of *H. dromedarii* were collected from the anus and 33.8% from the sternum.

**Figure 5:**
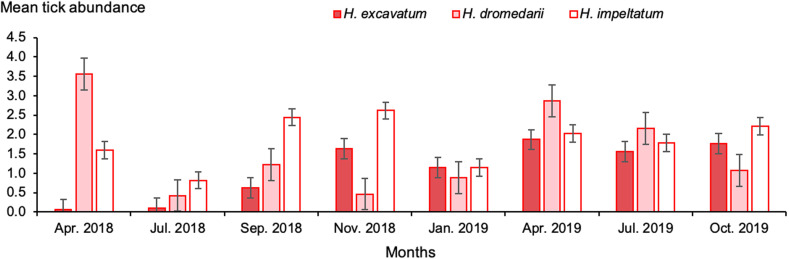
Monthly mean abundance of one-humped camels with *Hyalomma* ticks during the study period in southern Tunisia. Bars: standard error.

The three main *Hyalomma* species were collected throughout the whole sampling period with seasonal variation in the abundance of each species. Highest infestation abundance with *H. dromedarii* was reported in April 2018 (3.6 ticks/animal) and the lowest was 0.4 ticks/animal in November. Highest infestation abundance by *H. excavatum* (1.9 ticks/animal) occurred in April 2019 and the lowest was in April and July 2018, while highest infestation abundance by *H. impeltatum* was recorded in November 2018 (2.6 ticks/animal) and the lowest was in July 2018 (Fig. 5, Supplementary Table S1).

**Figure 6:**
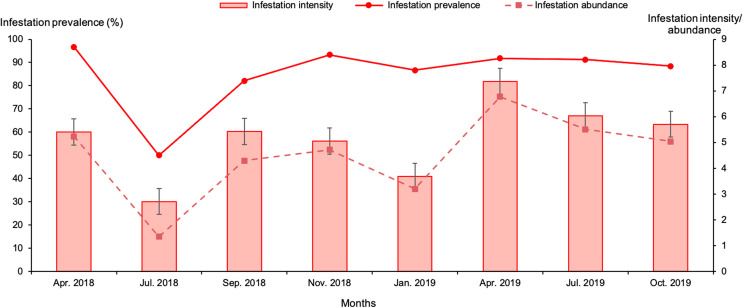
Monthly infestation prevalence, intensity and abundance of camels with *Hyalomma* spp. during the study period in Tataouine. Bars: standard error.

The highest overall infestation prevalence was recorded in April 2018 (96.7%) and the lowest in July 2018 (50%) (*p* < 0.01). Infestation intensity varied between 2.7 and 7.4 ticks per animal (Fig. 6). Despite the difference in total number of examined camels between the beginning of the study (around 30 animals examined in April and July 2018) and later on (43–67 camels were examined between September 2018 and October 2019), the infestation prevalence varied between 82.1% and 96.7% throughout the study period (except in July 2018 where fewer animals were examined).

**Figure 7:**
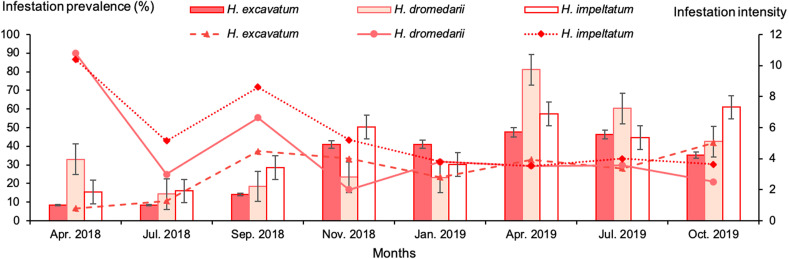
Monthly infestation prevalence and intensity of camels with *Hyalomma* species during the study period. Columns: infestation intensity; lines: infestation prevalence. Bars: standard error.

The three tick species were found on camels at all sampling dates. The mean infestation prevalence by *H. impeltatum*, *H. dromedarii* and *H. excavatum* was 46.2, 37.4 and 26.2%, respectively. The animals were infested by one or two tick species and rarely by the three species. The highest infestation prevalence by *H. dromedarii* (90%) and *H. impeltatum* (86.7%) occurred in April 2018 (Fig. 7). The mean infestation intensity was nearly similar for *H. impeltatum* (4.6 ticks/animal) and *H. dromedarii* (4.4 ticks/animal), but somewhat lower for *H. excavatum* (3.6 ticks/animal) (*p* = 0.9). The highest infestation intensity was reported for *H. dromedarii* in April 2019 (9.7 ticks/animal) and the lowest was observed for *H. excavatum* (1 tick/animal) in April 2018 (Fig. 7).

All age categories of camels were infested by ticks, the overall infestation prevalence was between 82.7% and 97.4%, with no statistically significant difference (*p* = 0.1). A detailed description of the monthly adult *Hyalomma* tick infestation of one-humped camels in southern Tunisia according to age group is provided in Supplementary Table S1.

Although fewer male camels were examined, our results still show that females were significantly more at risk of tick infestation (88.3%) than males (65.5%) (*p* < 0.01) (Supplementary Table S1).

There was a significant difference between the infestation prevalence of camels according to the localities. The highest infestation prevalence was recorded in Remada (93.5%; *n* = 102/109) followed by El Ouara (92.6%; 113/122) then Tataouine North (80.4%; *n* = 41/51) and Dhiba (75.8%; 94/124) (*p* < 0.01).

No significant correlations were found between infestation indicators and the physical characteristics of the study sites such as Temperature (*r* = −0.53; *p* = 0.17), Relative Humidity (*r* = −0.42; *p* = 0.29), and NDVI (*r* = 0.22; *p* = 0.59).

### Molecular identification and *H. dromedarii* mitochondrial Cox1 and 16S rDNA sequence variation

**Figure 8:**
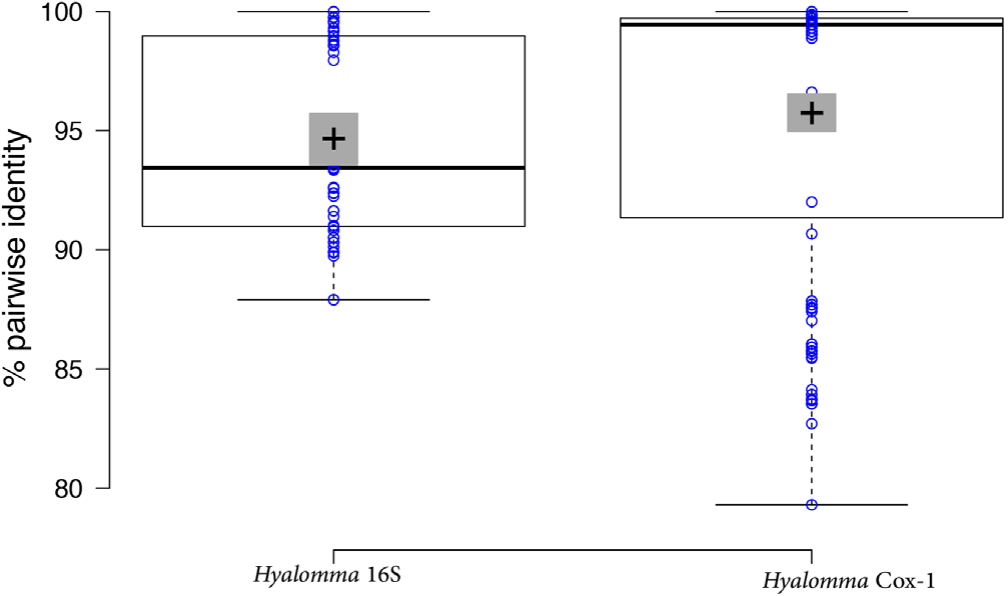
Pairwise percent identities among 16S and Cox1 genes in the *Hyalomma* species studied. Central lines show the medians; box limits indicate the 25th and 75th percentiles as determined by R software; whiskers extend 1.5 times the interquartile range from the 25th and 75th percentiles, outliers are represented by dots; crosses represent sample means; bars indicate 95% confidence intervals of the means; data points are plotted as open circles. *n* = 55, 231 sample points.

Genetic variation in a population of *H. dromedarii* from southern Tunisia was examined using mitochondrial Cox1 (850 bp) and 16S rDNA (455 bp) sequence data. Of the 42 Cox1 sequences generated during this study, 26 were unique at the nucleotide level. These unique Cox1 sequences have been deposited in GenBank under accession numbers: MT040954; MT062376, MT066414–MT066417; MT093505–MT093514; MT107481–MT107484; MT108550 and MT108551. The Cox1 nucleotide alignment dataset of *H. dromedarii* comprised 673 sites, and nucleotide substitutions were identified in 103 sites resulting in an average pairwise identity of 97% (Fig. 8). As regards sequence similarity to published *H. dromedarii* Cox1 sequences, 13 of these 26 unique Cox1 sequences exhibited 100% identity to Cox1 sequences from the Sinai area of Egypt and from Ethiopia. The other half of our unique Cox1 sequences were not 100% identical to any known *H. dromedarii* sequences and were therefore considered novel (Supplementary Table S2). One Cox1 Tunisian sequence shared 100% identity with an Ethiopian *H. dromedarii* tick (identified in Ethiopia as a natural hybrid of *Hyalomma rufipes*).

As regards *H. dromedarii* 16S rDNA sequence variation, our analysis revealed 11 unique sequences from the present study that were submitted to GenBank under accession numbers MN960579–MN960590. Nucleotide substitutions were identified at 78 sites resulting in an average pairwise identity of 95%. However, in contrast to the Cox1 sequences, only two of the 11 unique 16S rDNA sequences were identical to previously described sequences (Supplementary Table S2). In general, our BLAST search showed that the Tunisian *H. dromedarii* Cox1 and 16S sequences were most similar to those from Egypt, but also to those obtained in other countries such as Senegal, Ethiopia and Saudi Arabia, collected mainly from camels but some also from gerbil (*Dipodillus dasyurus*) in Saudi Arabia (Supplementary Table S2).

### Phylogenetic relationship

**Figure 9:**
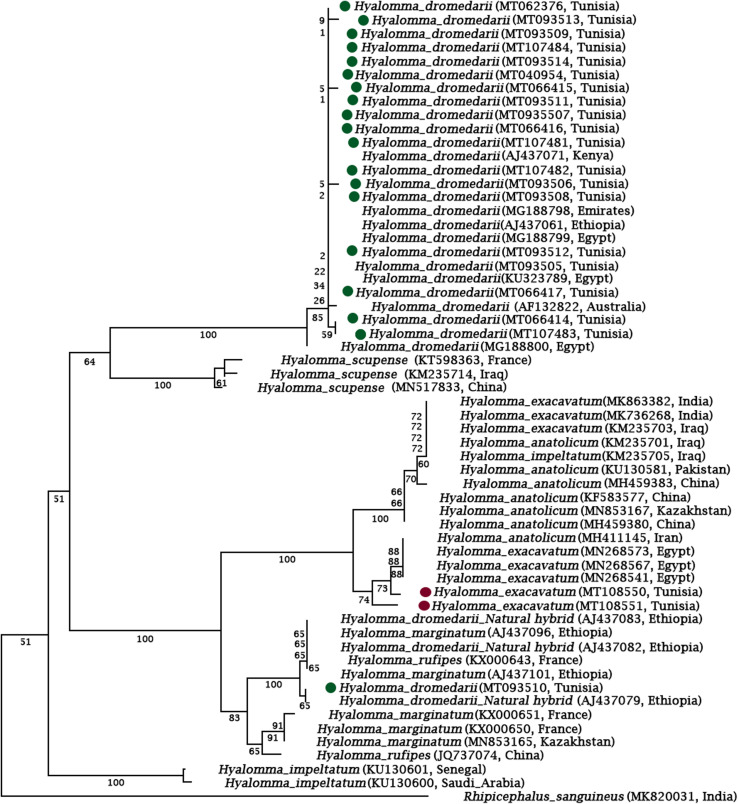
Maximum likelihood tree depicting the phylogenetic relationships of *H. dromedarii* mitochondrial Cox1 sequences. Cox1 sequence from *Rhipicephalus sanguineus sensu lato* is included as an outgroup. Numbers at the branches refer to bootstrap values (percentages) based on 1000 replicates, and the branch lengths are drawn to scale.

Phylogenetic analysis resolved the *H. dromedarii* Cox1 sequences into two evolutionary lineages (Fig. 9). Nearly all the Tunisian sequences belonged to the first lineage, which also included sequences from Egypt, United Arab Emirates, Ethiopia, Kenya and Australia. The second lineage comprised three sequences from Tunisia and three from Ethiopia.

**Figure 10:**
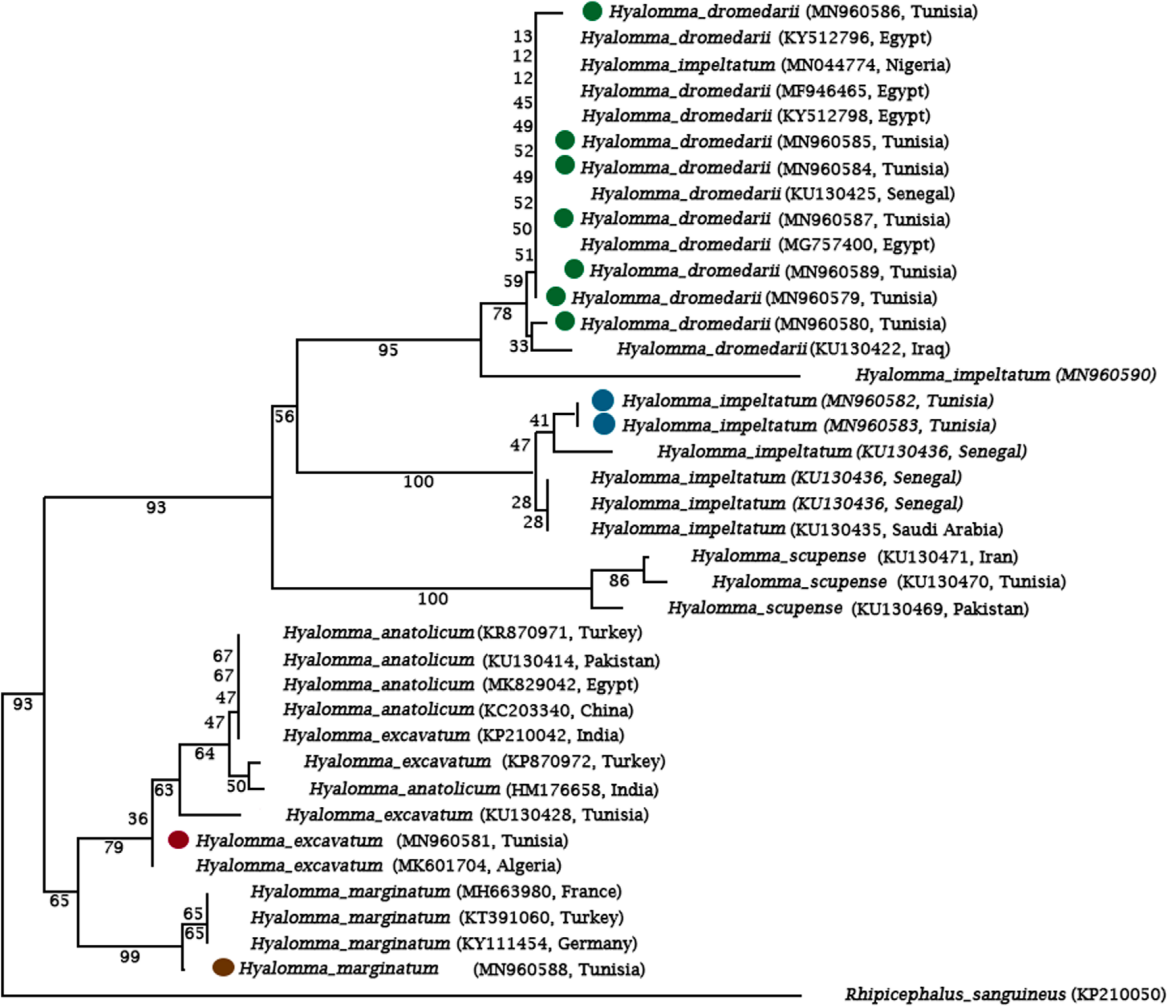
Maximum likelihood tree depicting the phylogenetic relationships of *H. dromedarii* 16S rDNA sequences. Cox1 sequence from *Rhipicephalus sanguineus sensu lato* is included as an outgroup. Numbers at the branches refer to bootstrap values (percentages) based on 1000 replicates, and the branch lengths are drawn to scale.

For 16S phylogeny, sequences from *H. impeltatum* and *H. excavatum* from this study were also included (Fig. 10). In common with the Cox1 phylogenies, the lineages based on the 16S sequences are comprised of sequences from different countries.

## Discussion

The present study aimed at understanding the seasonal activity of three *Hyalomma* species (*Hyalomma dromedarii, Hyalomma impeltatum* and *Hyalomma excavatum*) infesting camels in extensive production systems in the Saharan bioclimatic zone in southern Tunisia. The preferred attachment sites and the influence of environmental factors on tick infestation were also investigated. Despite the low observed infestation intensity compared to other studies, our results revealed that camels are permanently infested by ticks that may lead to possible infections by different pathogens. Given the particular significance of *H. dromedarii* not only in this region but also its large distribution in other bioclimatic zones in Tunisia and North Africa, we also inferred the phylogenetic diversity of this tick species, as well as other *Hyalomma* species, based on the mitochondrial Cox1 and 16S genes which suggest the presence of gene flow.

Our 18-month tick survey involving collection of 1902 adult ixodid ticks from 406 one-humped camels in southern Tunisia revealed the presence of four tick species belonging to the genus *Hyalomma* (41.1% *H. impeltatum,* 32.9% *H. dromedarii,* 25.9% *H. excavatum* and a single specimen of *H. marginatum*). A comparable study conducted on camels for one year in a hot and dry area in Iran showed that *H. dromedarii* was the most abundant species (84.7%), followed by *H. marginatum* (8.7%), *H. excavatum* (5.4%), and *H. anatolicum* (1.2%) [19]. A similar survey undertaken in Sudan on adult ticks infesting camels for two consecutive years revealed a high abundance of *H. dromedarii* (88.9%), followed by *H. impeltatum* (7.7%) and *H. anatolicum* (3.3%) [18]. Taken together, these findings confirm that *H. dromedarii* is the most common and dominant tick species infesting camels in arid areas from different countries in north and eastern Africa and the Middle East.

Like for all tick species, the composition of tick fauna on camels in different regions is influenced by several factors including, but not restricted to, the ecological conditions, the type of agricultural system, and the sampling period [5, 18, 19, 42, 55]. For example, previous surveys carried out in semi-intensive production systems, in which camels were kept together with other livestock species and under different climatic conditions, showed that other tick species not typical to camels can also infest these animals [24, 31]. This is illustrated by a study conducted in Central Tunisia from April 2011 to March 2012 in an area characterized by an arid climate and a dominance of halophyte pastures, revealing the presence on camels of *H. impeltatum* (53%) and *H. dromedarii* (45%), but also a few adult *Rhipicephalus turanicus* specimens (0.03%) [21]. Another example is a survey performed in south-western Tunisia (Tozeur district) where one-humped camels are kept together with cattle, which revealed the presence of *H. dromedarii* (82%), *H. impeltatum* (15%), *H. marginatum* (2.3%), a few *H. scupense* specimens (0.3%), and a single *R. sanguineus sensu lato* specimen [31]. *Hyalomma scupense* has also been reported on camels from Iran [32]. In a high-altitude area in Ethiopia, and due to the close contact of camels with cattle, *Rhipicephalus* and *Amblyomma* ticks were more abundant than *Hyalomma* ticks as the environment is more suitable for this species [55]. The presence of ticks from the *Rhipicephalus sanguineus* group is likely due to the cohabitation of camels with small ruminants and dogs, as shown in a survey carried out in Saudi Arabia where dogs were occasionally infested with *Hyalomma* ticks, such as *H. dromedarii, H. impeltatum* and *H. excavatum*, and camels were parasitized by *H. dromedarii* and *R. sanguineus sensu lato* [13]. The presence of *H. scupense* (the natural vector of *T. annulata* in cattle in Tunisia) outside of its known area of distribution may be due to the movement of infested cattle from the northern to the southern parts of Tunisia [31].

There were more *H. dromedarii* and *H. excavatum* male ticks than females (sex ratio M:F was 1.2 and 1.5, respectively). Our results are consistent with previous reports in the Middle East that showed higher numbers of male *H. dromedarii* ticks than females. This is not surprising since females take a shorter time to feed on camels before they detach from the animals to lay eggs, while males can attach for a longer period and mate with several females [36]. This was not the case for *H. impeltatum* where more females than males were collected: this could be explained by sampling fluctuations.

Our finding that the sternum and anus followed by the udder and inner thigh were the preferred tick attachment sites on camels is in agreement with a previous observation by Elghali and Hassan in 2009 [18]. This pattern could be explained by the physiological status of camels since the anus is less affected by diurnal variation of body temperature [30] and these body parts are not accessible to grooming. In these regions, the skin is thinner as observed in cattle with *H. scupense* [20, 30].

As regards seasonal activity of the ticks, our survey showed that adults of all *Hyalomma* species (except *H. marginatum*) were found during the whole study period which may suggest that these species are multivoltine.

Lower total tick numbers were collected by Gharbi et al. in 2013 [21] in Central Tunisia and the abundance varied between 0.05 and 2.6 ticks/animal for each tick species, while the abundance values recorded in our study varied between 0.5 and 3.6 ticks/animal. Differences in tick numbers collected could be related to the study designs since in Central Tunisia, only one herd of 30 heads of one-humped camels was monitored for adult tick infestation, while in the current study, more than 10 randomly selected herds totalling 406 heads in their natural Saharan climatic zone were surveyed.

Camels in Tunisia are not massively infested by ticks in comparison to other studies carried out under similar climatic conditions where camels were shown to be more infested with ticks throughout the year in Egypt (tick abundance reached 173 ticks/animal) [49], Emirates (mean tick abundance was 18.52 ticks/animal) [36] and Kenya (a total of 31,040 ticks was collected and the mean tick number varied between 40 and 150 ticks/per camel, which can lead to anemia and calf mortality) [15]. The low infestation intensity observed in the present study could be explained by the low presence of ticks in the environment of camels and to differences in herd characteristics and management.

The infestation prevalence varied significantly according to month and higher tick burden was recorded in April 2019. This monthly difference in tick infestation could be explained by the increase of temperature and low relative humidity during spring and summer, leading to higher tick burden in hot seasons and it could be attributed to the difference of examined herds and localities. This is consistent with previous studies performed in Iran [32] and Ethiopia [55].

No significant correlations were found between tick infestation and NDVI, LST and RH%. This could be explained by the distance between studied regions since they belong to the same district and have nearly the same characteristics. This result is consistent with previous findings from the United Arab Emirates [36].

We also noted that female camels were more infested with ticks than males, this finding is consistent with the observations of Elghali and Hassan in 2009 [18] who also hypothesised that lactation and pregnancy stresses may render females more susceptible to tick infestation. By contrast, age had no effect on tick infestation in the present study. This finding is in agreement with comparable work undertaken in Ethiopia [29]. However, this is in contrast to a previous survey from Central Tunisia, in which a positive correlation between camels’ age and tick burdens was observed [21].

DNA from ticks identified as *H. dromedarii* by morphology [52] was amplified and the morphology-based species designations were validated by sequencing the mitochondrial cytochrome oxidase subunit I (Cox1) and 16S genes. It is worth mentioning that only a minority of the Cox1 and 16S sequences that we generated were very similar to published sequences in public databases, whereas the majority were novel.

Phylogenetic reconstruction using the Cox1 and 16S sequence data that we generated during this study and previously published Cox1 and 16S sequences enabled us to draw two conclusions regarding the relatedness of *H. dromedarii* in southern Tunisia. First, the tree topologies resulting from maximum likelihood inference provide support for the co-existence of two distinct genetic lineages within the species in southern Tunisia. This reflects, within the confines of a single geographical site, the full extent of the known *H. dromedarii* diversity identified in northern and western Africa and also in Asia. This is because several Cox1 and 16S sequences have been published in these regions, and analyses of the relationships between these sequence results are similar to the topology defined by two distinct evolutionary lineages that co-exist in southern Tunisia. Second, the clades in our phylogenies have no correlation with geographical origin; indeed, there is extensive intermingling regardless of sampling location. This implies that the divergent lineages have evolved independently. Previous studies suggested that the divergence of lineages and the diversity of the *Hyalomma* genus is related to host movements, biogeographic separation, tectonic events, and changes in the environment [42].

In the present work, the molecular analyses of *Hyalomma* Cox-1 put *H. impeltatum* and *H. dromedarii* in the same clade as reported before [42], while *H. excavatum* was distant from *H. dromedarii* (Fig. 10). This is not surprising given the complexity of morphological distinctions between *H. dromedarii* and *H. impeltatum* [3] which could at least be partly attributable to possible hybridization that may occur in mitochondrial genes [38].

Previous molecular studies showed that some *H. dromedarii* specimens were indistinguishable from *H. rufipes*, and analysis of the internal transcribed spacer (ITS) and cytochrome genes suggest the occurrence of gene flow among these two species. Whether the fact that the one Tunisian Cox1 sequence in this study shared 100% identity with *H. dromedarii* (a natural hybrid of *Hyalomma rufipes* from Ethiopia) [38] is an illustration of such gene flow is beyond the scope of this study.

## Conclusion

The data presented herein confirm that *H. dromedarii* and *H. impeltatum* are the most abundant tick species associated with camels in Saharan climates, as exemplified by southern Tunisia. These infestations do not exhibit strong seasonal variations but are instead high throughout the year. This high year-round infestation implies either a lack or inadequacy of measures currently applied to control the ticks. Besides the effects on animal health and constraints on productivity, one of the possible consequences of such levels of tick infestation is the risk of the emergence of zoonotic diseases such as CCHF (in another study using the same samples collected for our study, a large proportion of the camels were positive for CCHFv by serology and one tick specimen was also infected by CCHFv [8]). There is an urgent need for regular and intensive application of measures intended to control tick infestation of camels in Southern Tunisia.

The potential presence of natural hybrids among *H. dromedarii* implies the need for more investigations into the genetic structure of *Hyalomma* species in Tunisia. The behaviour of off-host stages and tick competition in the natural environment and their interaction with their hosts are also worthy of further investigations.

## Conflict of interests

The authors declare that they have no competing interests.

## Supplementary Material

The supplementary material of this article is available at https://www.parasite-journal.org/10.1051/parasite/2021038. ***Table S1***. Monthly adult *Hyalomma* tick infestation of camels in Southern Tunisia according to age group and gender.
***Table S2***. BLASTn search results for 16S and Cox-1 genotype information for unique sequences.
