# Supplementary material for: Phenology and phylogeny of Hyalomma spp. ticks infesting one-humped camels (Camelus dromedarius) in the Tunisian Saharan bioclimatic zone
Source: Parasite. 2021 May 18;28:44. doi: 10.1051/parasite/2021038 (PMC8132600; doi:10.1051/parasite/2021038)
Supplement: Supplementary file 1 — Table S1. Monthly adult Hyalomma tick infestation of camels in Southern Tunisia according to age group and gender. Table S2. BLASTn search results for 16S and Cox-1 genotype information for unique sequences. [file parasite-28-44-s1.pdf]

Indicator	Risk factor	Apr. 2018	Jul. 2018	Sep. 2018	Nov. 2018	Jan. 2019	Apr. 2019	Jul. 2019	Oct. 2019	Overall	P
**Infestation prevalence (%±SE) (no. infested camels / no. examined camels**	*Age (Years)*										0.1
	≤2	9/9 (100)	4/7 (57.1±36)	2/6 (33.3)	0.0	10/10 (100)	17/19 (89.5±13)	23/26 (88.4)	11/11 (100)	76/88 (85.4)	
	]2-5]	1/1 (100)	1/2 (50±69)	1/3 (33.3±53)	0.0	16/21 (76.2±18)	8/9 (88.9±20)	7/7 (100)	0	34/44 (77.3±12)	
	]5-10]	4/4 (100)	5/6 (83.3±29)	26/31 (83.9±13)	5/7 (71.4±)	20/22 (90.9±12)	11/11 (100)	10/10 (100)	8/8 (100)	89/99 (89.9±6)	
	]10-15]	13/14 (92.9±13)	3/8 (37.5±33)	24/27 (88.9±12)	10/11 (90.9±17)	4/5 (80±35)	10/10 (100)	7/7 (100)	10/10 (100)	81/92 (88±6.6)	
	]15-20]	2/2 (100)	1/4 (25±42)	0	12/12 (100)	2/2 (100)	10/11 (90.9±17)	7/7 (100)	4/6 (66.7±37)	38/44 (86.4±10)	
	>20	0	0	0	29/30 (96.7±6)	0	1/1 (100)	0	7/7 (100)	37/38 (97.4±5)	
	Gender										<0.01
	Male	0/1	3/5 (60±43)	4/6 (66.7±37)	0	0	3/5 (60±43)	9/12 (75±24)	0	19/29 (65.5±17)	
	Female	29/29 (100)	11/23 (27.3±20)	51/61 (83.6±9.3)	56/60 (93.3±6.3)	52/60 (86.7±8.6)	53/56 (94.6±5.9)	43/45 (95.6±6)	38/43 (88.4±9.6)	333/377 (88.3±3.2)	
	Overall	29/30 (96.7±6.4)	14/28 (50±18.5)	55/67 (82.1±9.7)	56/60 (93.3±6.3)	52/60 (86.7±8.6)	57/61 (93.4±6.2)	54/57 (94.7±5.8)	40/43 (93±7.6)		
**Infestation intensity (no. ticks / no. infested camels)**	*Age (Years)*										
	≤2	7.1 (64/9)	2.3 (9/4)	3 (6/2)	0.0	5 (50/10)	5.8 (98/17)	5.1 (117/23)	5.4 (59/11)	403/76	
	]2-5]	5 (5/1)	2 (2/1)	6 (6/1)	0.0	3.8 (61/16)	5.8 (46/8)	6.7 (47/7)	0.0	4.9 (167/34)	
	]5-10]	3.3 (13/4)	2.4 (12/5)	6.3 (163/26)	6.4 (32/5)	3.3 (65/20)	7.4 (81/11)	7 (70/10)	6 (48/8)	5.4 (484/89)	
	]10-15]	5.2 (67/13)	4.7 (14/3)	4.7 (113/24)	5.6 (56/10)	2.5 (10/4)	7.2 (72/10)	5.7 (40/7)	6 (60/10)	5.3 (432/81)	
	]15-20]	4 (8/2)	1 (1/1)	0	4.3 (52/12)	3 (6/2)	11 (110/10)	5.7 (40/7)	4.8 (19/4)	6.2 (236/38)	
	>20	0	0	0	4.9 (143/29)	0	6 (6/1)	0	4.4 (31/7)	4.9 (180/37)	
	**Gender**										
	Male	0	3.3 (10/3)	1.5 (6/4)	0	0	4.6 (14/3)	4.3 (39/9)	0	3.6 (69/19)	
	Female	5.4 (157/29)	2.5 (28/11)	5.5 (282/51)	5 (283/56)	3.7 (192/52)	7.5 (399/53)	6.4 (275/43)	5.4 (217/40)	5.5 (1833/333)	
	Overall	5.4 (157/29)	2.7 (38/14)	5.2 (288/55)	5 (283/56)	3.7 (192/52)	7.2 (413/57)	5.8 (314/54)	5.4 (217/40)		

**Tick abundance (no. tick species/no. examined animals**	***H. impeltatum***	1.6 (48/30)	0.8 (23/28)	2.4 (164/67)	2.6 (157/60)	1.1 (69/60)	2 (124/61)	1.7 (102/57)	2.2 (95/43)	1.9 (782/406)
	H. dromedarii	3.5 (107/30)	0.4 (12/28)	1.2 (82/67)	0.46 (28/60)	0.9 (53/60)	2.9 (175/61)	2.1 (123/57)	1.1 (46/43)	1.5 (626/406)
	H. excavatum	0.07 (2/30)	0.1 (3/28)	0.6 (42/67)	1.6 (98/60)	1.1 (69/60)	1.9 (114/61)	1.6 (89/57)	1.8 (76/43)	1.2 (493/406)

Table 1: Monthly adult *Hyalomma* tick infestation of camels in Southern Tunisia according to age group and gender

Table 2: BLASTn search results for 16S and Cox-1 genotype information for unique sequences

	16S						Cox-1				
**Sample N°**	***Collection date***	***BLAST hit***		***Tick species***	***Country***	***Host***	***BLAST hit***		***Tick species***	***Country (district)***	***Host***
		Accession	***% identity***				***Accession***	***% identity***			
Tik1	27/09/18	MG757400.1	99.55	*H. dromedarii*	Egypt	Camels	KU323789.1	100	*H. dromedarii*	Egypt (Sinai)	Camel
Tik2	25/09/18	MG757400.1	99.75	*H. dromedarii*	Egypt	Camels	KU323789.1	99.17	*H. dromedarii*	Egypt (Sinai)	Camel
Tik4	25/09/18	MG757400.1	99.53	*H. dromedarii*	Egypt	Camels	KU323789.1	99.86	*H. dromedarii*	Egypt (Sinai)	Camel
Tik5	11/07/18	KY945490.1	99.77	*H. dromedarii*	Egypt	Camels	KU323789.1	100.00	*H. dromedarii*	Egypt (Sinai)	Camel
Tik7	27/09/18	KU130425.1	99.51	*H. dromedarii*	Senegal	NA	KU323789.1	99.87	*H. dromedarii*	Egypt (Sinai)	Camel
Tick8	27/09/18	KR870971.1	97.37	*H. excavatum*	Turkey	NA	MK863382.1	97.54	*H. excavatum*	India	Cattle
Tik9	27/09/18	MG757400.1	99.32	*H. dromedarii*	Egypt	Camels	KU323789.1	100	*H. dromedarii*	Egypt (Sinai)	Camel
Tik10	25/09/18	MG757400.1	99.32	*H. dromedarii*	Egypt	Camels	AJ437061.1	100	*H. dromedarii*	Ethiopia	Camel
Tick11	10/04/19	KU130436.1	99.51	*H. impeltatum*	Senegal	NA	NA	NA	NA	NA	NA
Tick13	10/04/19	KU130436.1	99.51	*H. impeltatum*	Senegal	NA	NA	NA	NA	NA	NA
Tik16	27/11/18	MG757400.1	99.77	*H. dromedarii*	Egypt	Camels	AJ437061.1	100	*H. dromedarii*	Ethiopia	Camel
Tik17	26/11/18	MG757400.1	99.32	*H. dromedarii*	Egypt	Camels	KU323789.1	99.73	*H. dromedarii*	Egypt (Sinai)	Camel
Tik23	27/09/18	KU130425.1	99.27	*H. dromedarii*	Senegal	NA	KU323789.1	99.86	*H. dromedarii*	Egypt (Sinai)	Camel
Tik34	22/01/19	MG757400.1	98.87	*H. dromedarii*	Egypt	Camels	AJ437061.1	99.84	*H. dromedarii*	Ethiopia	Camel
Tik40	26/11/18	KY512796.1	95.54	*H. dromedarii*	Egypt	Camels	KU323789.1	100	*H. dromedarii*	Egypt (Sinai)	Camel
Tik41	22/01/19	MG757400.1	97.64	*H. dromedarii*	Egypt	Camels	KU323789.1	100	*H. dromedarii*	Egypt (Sinai)	Camel
Tik43	22/01/19	KY512798.1	93.82	*H. dromedarii*	Egypt	*Dipodillus dasyurus*	KU323789.1	99.74	*H. dromedarii*	Egypt (Sinai)	Camel
Tik46	22/01/19	KY512798.1	95.64	*H. dromedarii*	Egypt	*Dipodillus dasyurus*	KU323789.1	99.73	*H. dromedarii*	Egypt (Sinai)	Camel
Tik48	22/01/19	MG757400.1	99.77	*H. dromedarii*	Egypt	Camels	KU323789.1	100	*H. dromedarii*	Egypt (Sinai)	Camel
Tick57*	26/11/18	MK058362.1	100	*H. marginatum s. l*	Greece	avian	AJ437079.1	100	*H. dromedarii*	Ethiopia	Camel
Tik60	10/04/19	MG757400.1	99.77	*H. dromedarii*	Egypt	Camels	KU323789.1	100	*H. dromedarii*	Egypt (Sinai)	Camel
Tik74	10/04/19	KU130425.1	99.76	*H. dromedarii*	Senegal	Camels	KU323789.1	100	*H. dromedarii*	Egypt (Sinai)	Camel

Tik76	10/04/19	KY945490.1	99.77	*H. dromedarii*	Egypt	Camels	KU323789.1	100	*H. dromedarii*	Egypt (Sinai)	Camel
Tik78	10/04/19	MG757400.1	99.77	*H. dromedarii*	Egypt	Camels	AJ437061.1	99.86	*H. dromedarii*	Ethiopia	Camel
Tik85	10/04/19	MG757400.1	100.00	*H. dromedarii*	Egypt	Camels	AJ437061.1	100	*H. dromedarii*	Ethiopia	Camel
Tik86	10/04/19	MG757400.1	98.76	*H. dromedarii*	Egypt	Camels	KU323789.1	99.86	*H. dromedarii*	Egypt (Sinai)	Camel
T104	17/10/19			NA			KU323789.1	99.70	*H. dromedarii*	Egypt (Sinai)	Camel
T105	17/1019			NA			AJ437061.1	99.86	*H. dromedarii*	Ethiopia	Camel

* Tick 57: identified as *Hyalomma dromedarii* by morphology and seems to be a hybrid of *Hyalomma rufipes* by molecular analyses
NA= Not available
